# Determination of the preliminary discriminating concentration of broflanilide against malaria vector mosquito *Anopheles gambiae* by multi-centre susceptibility testing

**DOI:** 10.1186/s12936-025-05728-5

**Published:** 2026-03-09

**Authors:** Yuki Ando, Kunizo Mori, John Bradley, Janneke Snetselaar, Graham Small

**Affiliations:** 1 Mitsui Chemicals Crop & Life Solutions, Inc., Tokyo, Japan; 2 Advisor to Mitsui Chemicals Crop & Life Solutions, Inc., Tokyo, Japan; 3 London School of Hygiene and Tropical Medicine, London, UK; 4 Innovative Vector Control Consortium, Liverpool, UK

**Keywords:** Mosquito control, Indoor residual spraying, Broflanilide, Discriminating concentration

## Abstract

**Background:**

Indoor residual spraying (IRS) of insecticides is widely used as an effective method to control malaria vector mosquitoes in sub-Saharan Africa. In 2023, a new IRS product, VECTRON™ T500 (Mitsui Chemicals Crop & Life Solutions, Inc.), was launched. This product contains broflanilide, a novel active ingredient for IRS, and has been confirmed to exhibit long-lasting insecticidal efficacy against malaria vector mosquitoes. However, the discriminating concentration to assess the susceptibility of wild *Anopheles* populations to broflanilide has not yet been determined.

**Methods:**

In this study, WHO bottle bioassays were conducted in nine research facilities to collect dose response data on broflanilide against adult female mosquitoes of the insecticide susceptible *Anopheles gambiae s.s.* Kisumu strain. These data were statistically analysed and validated following WHO guidelines.

**Results:**

It was determined that the preliminary discriminating concentration of broflanilide for *An. gambiae* mosquitoes should be 18 μg/bottle.

**Conclusions:**

In this study, the preliminary discriminating concentration of broflanilide for *An. gambiae* mosquitoes was determined. The results of this study will provide a useful benchmark for susceptibility monitoring of wild mosquito populations in regions of sub-Saharan Africa into which VECTRON™ T500 is being introduced for malaria vector control.

## Background

Anopheline mosquitoes transmit malaria parasites to humans leading to malaria infections in sub-Saharan African countries [1]. To reduce and eliminate malaria in these countries, effective methods for the control of vector mosquitoes are essential. One of the most effective methods of mosquito vector control is indoor residual spraying (IRS) of insecticides, which has been widely used for several decades [2, 3]. Killing blood-fed mosquitoes that rest on the indoor walls of houses, reduces the local vector population and prevents mosquitoes that have taken an infected blood meal from transmitting the malaria parasites during subsequent blood feeding. It is recommended that IRS products should have long-lasting residual efficacy, preferably of at least 6 months duration, as this is generally the duration of the main period of malaria transmission in sub-Saharan Africa [1].

A number of insecticides with several different modes of action (e.g., DDT, carbamates, organophosphates, and pyrethroids) have been used previously for IRS malaria vector control programmes, but overuse of the products into which these insecticides have been formulated has led to the evolution and spread of insecticide resistance in many vector populations [2, 4–9]. This, in turn, has led to reduced effectiveness of some of the existing IRS products; therefore, the development and use of IRS products containing novel insecticides, to which there is currently no detected resistance, is essential to our fight against malaria [10–19].

VECTRON™ T500 (active ingredient broflanilide 50% wettable powder), developed by Mitsui Chemicals Crop & Life Solutions, Inc. (MCCLS), is a new IRS product that was evaluated and listed by the World Health Organization Prequalification Unit Vector Control Product Assessment Team (WHO PQT/VCP) in March 2023 and was released in December 2023. Previous studies have confirmed that this formulation exhibits high and long-term efficacy against Anopheline mosquitoes [20–28]. The active ingredient, broflanilide (tradename TENEBENAL^™^), is a novel meta-diamide insecticide compound developed by MCCLS. This compound is classified in IRAC group 30 (allosteric modulator of gamma-aminobutyric acid (GABA)-gated chloride ion channels) and has a novel mode of action [29, 30] that differs from the active ingredients in existing IRS formulations. Previous studies have detected no cross-resistance to broflanilide in pyrethroid resistant mosquito strains or populations; therefore, the rotational use of this compound with compounds with different modes of action can be a viable option for controlling these malaria vectors and manage resistance development [20–28].

As part of any resistance management strategy, it is essential to establish the baseline susceptibility of wild mosquito populations in regions where the use of an insecticide is planned and to monitor for the possible development of resistance thereafter. For this purpose, insecticide susceptibility test methods have been developed which use a concentration of an insecticide that will discriminate between insecticide susceptible and resistant mosquitoes in terms of mortality (DC: Discriminating Concentration). For this susceptibility testing, the World Health Organization (WHO) recommends conducting a WHO bottle bioassay [31, 32] with the DC for insecticide being used in mosquito vector control being determined via multi-centre studies led by the WHO (e.g., the DCs of existing compounds for *Anopheles gambiae* are as follows: clothianidin, 4 µg/bottle; flupyradifurone, 60 µg/bottle; transfluthrin, 2 µg/bottle; and chlorfenapyr, 100 µg/bottle) [32, 33]. However, the DC of broflanilide has not been determined yet by WHO due to its novelty [32], while in African countries, there is a need for baseline susceptibility testing by implementing programmes as they introduce VECTRON™ T500 into their IRS campaigns [20–23, 34–37]. Therefore, it is important to identify early on a preliminary DC (PDC) that can be used to monitor mosquito population susceptibility to broflanilide ahead of the introduction of this new insecticide and before the WHO publishes its definitive DC.

In this study to determine the PDC of broflanilide, the same process for DC establishment as described by the WHO [32] was followed as far as possible. Previous studies have shown that the addition of the adjuvant Mero^®^ (81% rapeseed oil methyl ester; Bayer AG, Crop Science Division) can enhance and stabilize the compound's efficacy by inhibiting crystallization [31–33, 38], and the DCs of broflanilide with 800 ppm Mero for *An. gambiae* have been proposed for use in association with large-scale community trials on VECTRON™ T500 in previous studies (6 μg/bottle and 17.82 μg/bottle) [34, 35]. However, these PDCs were estimated using only one or a few datasets from dose response tests with *An. gambiae*. According to WHO guidelines, it is desirable to have three or more datasets to establish the DC [32]. Therefore, this study aimed to propose a more robust PDC by generating broflanilide dose response data at multiple research facilities in Africa and the UK. Colonies of the insecticide susceptible *An. gambiae s.s.* Kisumu strain established at these research facilities were used for tests with a range of broflanilide concentrations and the data were statistically analysed to determine the PDC. This PDC will provide the necessary information for the stewardship of VECTRON™ T500 via broflanilide susceptibility monitoring at an early stage in the roll out of this IRS product.

## Methods

### Mosquito strain

Colonies of the *An. gambiae s.s.* Kisumu strain maintained by each trial facility were used in all the testing as an insecticide susceptible strain. This strain was originally collected in the Kisumu region of Kenya. All mosquitoes used were 2–5 day old, non-blood-fed adult females.

### Susceptibility testing with broflanilide by WHO bottle bioassays at multiple study facilities

To obtain data on the dose response to broflanilide of Kisumu strain mosquitoes, WHO bottle bioassays were conducted [31]. The glass bottles (250 mL, Wheaton bottles) were treated with 1 mL of broflanilide dissolved in a mixture of acetone and 800 ppm Mero. Bottles treated with acetone + 800 ppm Mero only were also prepared as a negative control. Twenty-five mosquitoes were introduced into each bottle and exposed for 1 h. After exposure, the mosquitoes were aspirated into clean cups and provided with cotton wool soaked in a 10% sugar solution ad libitum. Mortality was recorded at 24 h intervals up to 72 h post exposure. The bioassays were conducted under laboratory conditions (27 ± 2 ℃, 80 ± 10% RH).

There were some minor variations to the bioassays conducted by the 9 research facilities (i.e. the concentrations used, number of replicates and number of mosquitoes) which are detailed in Table 1. The data generated by the Liverpool Insect Testing Establishment (LITE) facility (UK) were fully Good Laboratory Practice (GLP) compliant. The data from the African research facilities were generated during the conduct of non-GLP semi-field experimental hut or community VECTRON^™^ T500 product registration trials following the standard operating procedures implemented at the respective facilities.

**Table 1:** Test conditions from each testing facility

Country (facility)	Location	Concentrations (μg/bottle)	Number of replicates	Number of mosquitos	Temperature (℃)	Humidity (%RH)
Zambia (Tropical Disease Research Centre)	Nchelenge	0.781, 1.563, 3.125, 6.25, 12.5, 25, 50	5	1037	23–28	48–70
Uganda (NMCD MOH)	Nosako	1.563, 3.125, 4, 6.25, 12.5, 25	4	750	27 ± 2	80 ± 10
DRC (University of Kinshasa)	Kinshasa	0.195, 0.39, 0.781, 1.563, 3.125, 6.25, 12.5, 25, 50	4	889	27 ± 2	80 ± 10
Burkina Faso (IRSS/DRO)	Vallée du Kou	0.781, 1.563, 3.125, 6.25, 12.5, 25, 50	4	778	27 ± 2	80 ± 10
Ghana (NMIMR)	Odumse	0.781, 1.563, 3.125, 6.25, 12.5, 25, 50	4	800	27 ± 2	80 ± 10
Benin (CREC/LSHTM)	Cotonou	0.05, 0.1, 0.25, 0.5, 1, 2.2, 4.6, 10	4	800	27 ± 2	75 ± 10
Tanzania (KCMUCo-PAMVERC)	Moshi	0.01, 0.0215, 0.0464, 0.1, 0.215, 1.0, 2.15, 4.64, 10	6	1661	25–27	80
Tanzania (NIMR Amani Research Centre)	Muheza	0.01, 0.0215, 0.0464, 0.1, 0.215, 1.0, 2.15, 4.64, 10	4	850	25–27	80
UK (LITE)	Liverpool	0.3, 0.86, 1.42, 1.98, 2.54, 3.1	9	1571	26 ± 2	75 ± 10

Data from 4 of the studies have previously been published elsewhere (Tanzania KCMUCo and NIMR [34], Benin [35], Burkina Faso [37]) but were included in our statistical analysis to provide further supporting information for the setting of the PDC.

### Data analysis

All data analysis was undertaken using Stata 18. A probit model was used to estimate the lethal concentration that would induce 99% mortality in mosquitoes (LC_99_). The independent variable was the logarithm of the concentration of broflanilide. Abbott’s formula was used to correct mortality in test replicate for mortality in the negative controls (acetone and 800 ppm Mero only).

$${\mathrm{Corrected}}\,{\mathrm{mortality}}\, = \,\frac{{\left( {{{\% }}\,{\mathrm{observed}}\,{\mathrm{mortality}}\, - \,{{\% }}\,{\mathrm{control}}\,{\mathrm{mortality}}} \right)}}{{\left( {100\, - \,{{\% }}\,{\mathrm{control}}\,{\mathrm{mortality}}} \right)}}\, \times \,100$$

Confidence intervals were estimated using the bootstrap, which took uncertainty in the control mortality into account. For each probit regression, a p-value for the Pearson’s Chi-squared test (goodness of fit) was calculated.

## Results

### Dose response in test results for each facility

**Fig. 1:**
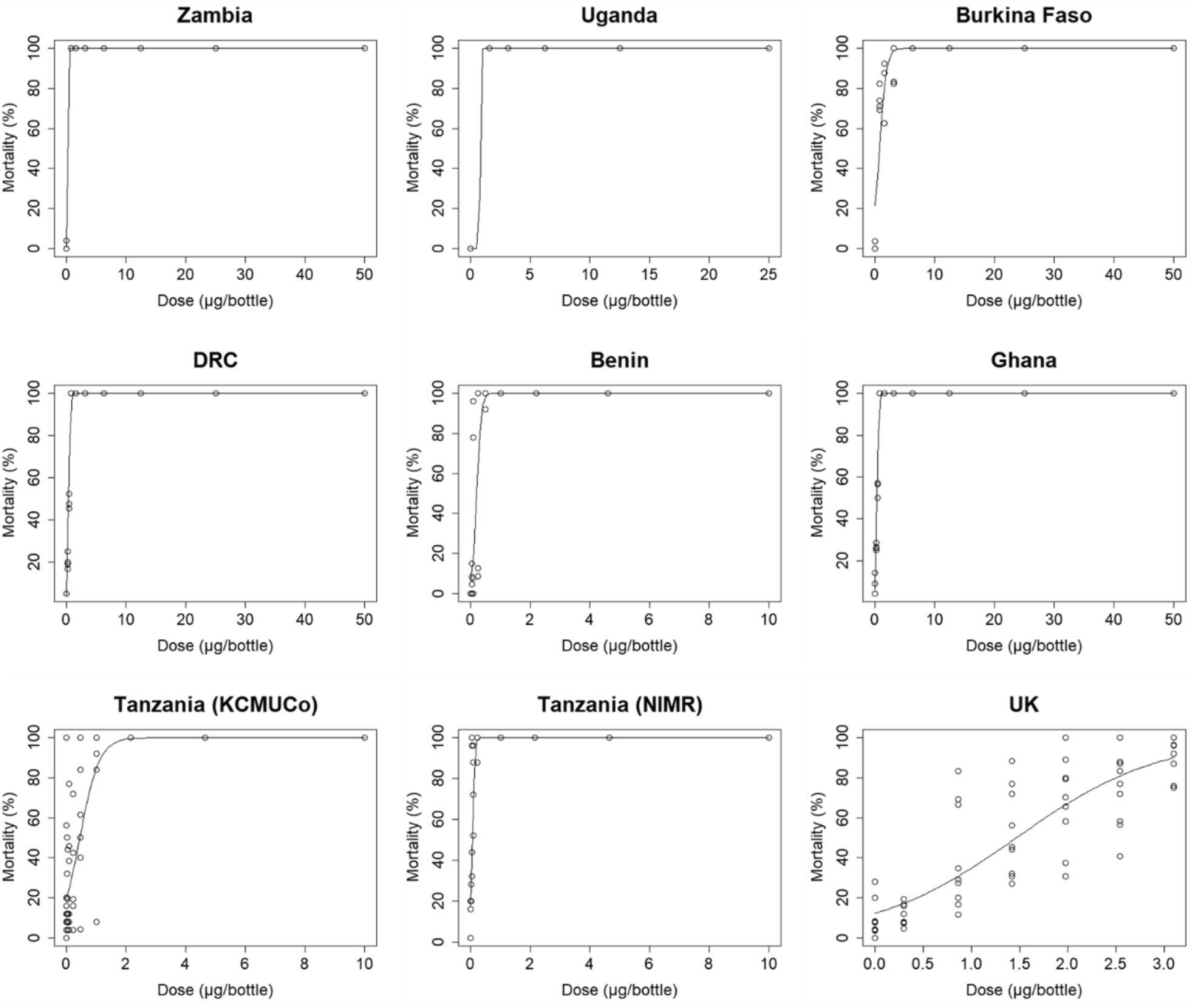
Dose response curves of broflanilide with the insecticide susceptible *An. gambiae* s.s. Kisumu strain

Figure 1 shows the dose–response curves of the susceptible *An. gambiae s.s.* Kisumu strain mosquitoes to broflanilide which were obtained from the WHO bottle bioassays conducted by the 9 research facilities. In eight of the datasets, excluding those from the UK facility, 100% mortality was confirmed within the tested concentration ranges, but there was variability in the lowest LC_100_ values (Zambia, DRC, and Ghana: 0.781 μg/bottle; Benin and Tanzania NIMR: 1.0 μg/bottle; Uganda: 1.562 μg/bottle; Tanzania KCMUCo: 2.15 μg/bottle; and Burkina Faso: 6.25 μg/bottle) (Table 2). Additionally, each dose–response curve rose very steeply which is typical for an insecticide susceptible strain (Fig. 1). In the bottle bioassay data from Zambia and Uganda, 100% mortality was observed at all broflanilide concentrations tested. Conversely, in the test results from the UK facility, the maximum mortality rate observed for the range of tested concentrations was 90.35%, and 100% mortality was not achieved.

**Table 2:** Summary of the data from multi-centre susceptibility testing of broflanilide against the insecticide susceptible *An. gambiae* s.s*.* Kisumu strain

Country	LC_99_ ± 95% CI (μg/bottle) [95% CI]	p-value of Chi-sq test	2 × LC_99_ (μg/bottle)	Number of valid datapoint	Observed lowest LC_100_ (μg/bottle)	2 × LC_100_ (μg/bottle)
Zambia	NA	NA	NA	0	0.781	1.562
Uganda	NA	NA	NA	0	1.563	3.126
Burkina Faso	8.42 [4.73–12.10]	0.3243	16.84	5	6.25	12.5
DRC	1.06 [0.91–1.21]	0.0636	2.12	2	0.781	1.562
Benin	1.02 [0.85–1.18]	0.0001	2.04	4	1	2
Ghana	1.08 [0.89–1.26]	0.0899	2.16	2	0.781	1.562
Tanzania (KCMUCo)	27.84 [17.75–37.90]	< 0.0001	55.68	7	2.15	4.3
Tanzania (NIMR)	0.57 [0.31–0.82]	0.4268	1.14	5	1	2
UK	13.44 [9.76–16.96]	0.0136	26.88	6	NA	NA

*CI* Confidence Interval

### Overview of estimated lethal concentration (LC_99_) values for each facility and determination of the preliminary discriminating concentration for broflanilide

The data and statistical analysis outputs from the nine dose response studies (LC_99_ and 95% CI, goodness of fit, number of data points with mortality rates neither 0% nor 100%, the lowest LC_100_ if available, and 2 × the LC99 or LC100) are summarized in Table 2. The LC_99_ of broflanilide for the susceptible *An. gambiae s.s.* Kisumu strain varied from 1.02 to 27.84 μg/bottle.

The guidance from WHO on the validation of datasets [32] considers several aspects. First, to be considered valid, each dataset should have at least two concentrations that killed < 50% of mosquitoes, one concentration that killed about 50%, two concentrations that killed > 50% of mosquitoes and one concentration that killed about 100% mosquitoes. After this, the validation of each dataset should consider the following: mortality of controls should be below the cut-off point of 20%; a minimum of six concentrations should be available to generate concentration–response curves and estimate lethal concentrations; the goodness of fit p-value should be > 0.05; and the lowest concentration that killed 100% of mosquitoes (LC_100_), when available. Considering the datasets generated by the 9 research facilities, none could satisfy all the validation requirements stated by the WHO. Therefore, the datasets which best matched WHO guidance were determined.

Generally, probit analysis represents the relationship between mortality and the logarithm of dose as a linear relationship [39]. Therefore, to perform linear regression, at least three data points are necessary in which the mortality rates of neither 0% nor 100% [if there are only two data points, the fit of the regression line will be perfect (R^2^ = 1)].The number of data points used in the probit analysis for calculating the LC_99_ was 0 or 2 in four datasets (Zambia, Uganda, DRC, and Ghana). Additionally, in three datasets, the p-value of the chi-squared test indicating the goodness of fit was less than 0.05 (Benin, Tanzania, and the UK).

There were two results with a goodness of fit p-value greater than 0.05 and three or more valid data points (Tanzania NIMR and Burkina Faso). The LC_99_ values were 0.57 μg/bottle (Tanzania NIMR) and 8.42 μg/bottle (Burkina Faso). Therefore, taking into account WHO guidance on determining the DC of insecticides for monitoring resistance in mosquitoes in which the highest LC_99_ is to be used [32], 2 × LC_99_ for the Burkina Faso dataset (16.84 μg/bottle) was taken to inform the appropriate PDC for susceptibility testing with broflanilide.

## Discussion

### Establishment of the preliminary discriminating concentration

Most of the dose response experiments were not specifically designed for the determination of the PDC for broflanilide but were part of non-GLP semi-field or community VECTRON™ T500 product registration trials. Therefore, it was not possible to meet all of the data validation criteria described by the WHO [32].

Based on the calculation of LC_99_ and statistical analysis results of each test shown in Table 2, the PDC of broflanilide for the susceptible *An. gambiae s.s.* Kisumu strain was determined. Following the verification method for data as guided by the WHO [32], for datasets with a goodness of fit *p* > 0.05 (Burkina Faso and Tanzania NIMR), the value twice the LC_99_ was adopted for those tests. For datasets with a goodness of fit p < 0.05 (Benin, and Tanzania KCMUCo), the reliability of the LC_99_ was regarded low. Additionally, the data with 100% mortality at all test concentrations (Zambia and Uganda) and the data where only two valid data points were obtained (DRC and Ghana), it was considered inappropriate to refer to the results of the probit analysis. For the results from the LITE facility in the UK, the goodness of fit p-value was less than 0.05 and no LC_100_ was confirmed.

Finally, among the nine LC_99_ values determined through the above data verification, the highest was 8.42 μg/bottle (Burkina Faso). According to the WHO guideline [32], twice this value, 16.84 μg/bottle, was determined to be the most appropriate for selecting the PDC for *An. gambiae*. Rounding this value to make it simpler results in 17 μg/bottle; however, since 17 is a prime number, it may pose difficulties in practical operations such as preparing stock solutions, working solutions, or performing serial dilutions. Additionally, several African countries intending to use VECTRON^™^ T500 are already conducting susceptibility tests with a provisional concentration of 18 μg/bottle (established from unpublished broflanilide dose response data). Following discussions with IRS programme implementers on the practicality of broflanilide susceptibility monitoring in sub-Saharan Africa and, therefore, to simplify the dilution of broflanilide for the treatment of bottles, the PDC for broflanilide of 18 μg/bottle (with 800 ppm Mero) is proposed.

### The variation between the estimated LC values among trial facilities

The results obtained in this research showed variations in the dose response of broflanilide for each test data set, even though the same strain (*An. gambiae s.s.* Kisumu) was used.

The mosquito colonies used in this study had been maintained and reared for a long time at the different testing facilities. Since the methods and conditions (e.g., temperature, humidity, density) for continuous rearing in each facility were not exactly the same, this might have led to variations in the general fitness and tolerance to exposure to broflanilide of the respective colonies. Ideally, cross-validation of the characteristics of the susceptible mosquito colonies and of the experimental methods should be conducted across testing facilities [32]. However, as mentioned previously, the eight bottle bioassays conducted in this study, apart from those conducted by the UK facility, were not specifically designed as dose–response studies to determine the PDC for broflanilide. Therefore, minor variations in the detailed procedures during the experiments (e.g., preparation and serial dilution of the broflanilide working solution, treatment of bottles, mosquito treatment when introducing into bottles or retrieval from bottles) or the holding conditions of mosquitoes after exposure to broflanilide in bottles (e.g., temperature, humidity, method of providing sugar solution) might also have influenced the dose–response to broflanilide in the mosquito colonies at each testing facility.

## Conclusion

In this study, nine datasets of WHO bottle bioassays conducted at the multiple trial facilities were conducted, the DC (2xLC_99_) of broflanilide for the *An. gambiae s.s.* Kisumu strain was determined to be 16.84 μg/bottle and a PDC of 18 μg/bottle is proposed for monitoring the susceptibility of wild populations of *An. gambiae* to broflanilide. Independently of the study described here, the U.S. President’s Malaria Initiative (PMI) Evolve Project and Abt Global performed an independent study using the same bottle bioassay methodology to assess the susceptibility of colonized and field sampled *An. gambiae s.s.* and *An. gambiae s.l.* to broflanilide in several sub-Saharan African countries. This study included the 18 µg/bottle concentration but also lower concentrations of 3, 6, and 9 µg/bottle. Almost complete mortality was observed in bottle bioassays with the 18 µg/bottle concentration, but lower mortality was observed in some tests with the lower concentrations (unpublished data). Therefore, data from this independent study support the adoption of the proposed preliminary discriminating concentration of broflanilide for *An. gambiae* mosquitoes of 18 μg/bottle.

There was considerable variability in the dose–response to broflanilide among the nine datasets used in this study. As a result of data validation, only two datasets were considered valid for the determination of the PDC. Data generated in further broflanilide dose–response studies are needed to establish a definitive DC for broflanilide, and the PDC proposed here may change based on the results of future studies sponsored by WHO to determine the definitive DC. However, for regions where the VECTRON™ T500 IRS product, containing broflanilide as an active ingredient, is already in use or will be used in the near future as part of malaria vector control campaigns, the PDC of broflanilide proposed by this study will serve as a benchmark for assessing the baseline susceptibility of wild populations of *An. gambiae* to broflanilide and to monitor for any changes in susceptibility with time. The data generated in susceptibility testing will facilitate rapid decision-making regarding the use of VECTRON™ T500 and, together with susceptibility data for insecticides used in other IRS products, will inform the implementation of insecticide resistance management through the rotation of IRS products containing insecticides with different modes of action.

## Data Availability

All data associated with this study are present in the paper. All other relevant data are available from the corresponding author upon reasonable request.

## Abbreviations

DC: Discriminating concentration
GLP: Good laboratory practice
IRS: Indoor residual spraying
LC: Lethal concentration
PDC: Preliminary discriminating concentration
